# Diagnostic performance of classification trees and hematological functions in hematologic disorders: an application of multidimensional scaling and cluster analysis

**DOI:** 10.1186/s12911-021-01678-5

**Published:** 2021-11-10

**Authors:** Fakher Rahim, Anoshirvan Kazemnejad, Mina Jahangiri, Amal Saki Malehi, Kimiya Gohari

**Affiliations:** 1 Research Center of Thalassemia and Hemoglobinopathy, Health Research Institute, Ahvaz Jundishapur University of Medical Sciences, Ahvaz, Iran; 2 Department of Biostatistics, Faculty of Medical Sciences, Tarbiat Modares University, Tehran, Iran; 3 Department of Biostatistics and Epidemiology, Faculty of Health, Ahvaz Jundishapur University of Medical Sciences, Ahvaz, Iran

**Keywords:** Diagnosis, Classification tree algorithms, Hematological discrimination indices, Iron deficiency anemia (IDA), β‐thalassemia trait (βTT), CRUISE tree algorithm, C5.0 tree algorithm

## Abstract

**Background:**

Several hematological indices have been already proposed to discriminate between iron deficiency anemia (IDA) and β‐thalassemia trait (βTT). This study compared the diagnostic performance of different hematological discrimination indices with decision trees and support vector machines, so as to discriminate IDA from βTT using multidimensional scaling and cluster analysis. In addition, decision trees were used to determine the diagnostic classification scheme of patients.

**Methods:**

Consisting of 1178 patients with hypochromic microcytic anemia (708 patients with βTT and 470 patients with IDA), this cross-sectional study compared the diagnostic performance of 43 hematological discrimination indices with classification tree algorithms and support vector machines in order to discriminate IDA from βTT. Moreover, multidimensional scaling and cluster analysis were used to identify the homogeneous subgroups of discrimination methods with similar performance.

**Results:**

All the classification tree algorithms except the LOTUS tree algorithm showed acceptable accuracy measures for discrimination between IDA and βTT in comparison with other hematological discrimination indices. The results indicated that the CRUISE and C5.0 tree algorithms had better diagnostic performance and efficiency among other discrimination methods. Moreover, the AUC of CRUISE and C5.0 tree algorithms indicated more precise classification with values of 0.940 and 0.999, indicating excellent diagnostic accuracy of such models. Moreover, the CRUISE and C5.0 tree algorithms showed that mean corpuscular volume can be considered as the main variable in discrimination between IDA and βTT.

**Conclusions:**

CRUISE and C5.0 tree algorithms as powerful methods in data mining techniques can be used to develop accurate differential methods along with other laboratory parameters for the discrimination of IDA and βTT. In addition, the multidimensional scaling method and cluster analysis can be considered as the most appropriate techniques to determine the discrimination indices with similar performance for future hematological studies.

**Supplementary Information:**

The online version contains supplementary material available at 10.1186/s12911-021-01678-5.

## Background

Microcytic anemia is the most common form of anemia, as a predominant hematologic disorder. IDA and βTT are the two common types of microcytic anemia disorders [1, 2]. The discrimination between IDA and βTT is a vital issue in hematology studies [3, 4]. IDA is a prevalent disorder worldwide, and βTT is, in turn, predominant in the Mediterranean region [5–10].

The discrimination between these two hematologic disorders is necessary to prevent iron overload and its complications caused by misdiagnosis and inaccurate treatment so as to determine the prenatal causes for hemoglobin chain disorders. However, the differential diagnosis of IDA from βTT is a major challenge given that they provide similar experimental conditions [3, 11, 12].

In addition to complete blood count (CBC), different tests have been already conducted to differentiate between IDA and βTT precisely; however, they are time-consuming and expensive. The definitive diagnostic methods for the IDA and βTT are respectively based on the increase in HbA2 (Hemoglobin A2), the increase in TIBC (total iron binding capacity), and also the decrease in serum iron and serum ferritin [4, 11, 13–16].

Due to the importance of discriminating between these types of anemia, various studies have been conducted since 1973 to identify appropriate, rapid, and low-cost differential indices for discriminating between IDA and βTT [17–41]. The existing gaps in the literature about hematological indices showed that each hematological index only includes one or some specific blood parameters. In addition, some indices like Nishad [33] and Matos and Carvalho [41] are suggested based on the parametric statistical model like the discriminant analysis. However, this parametric model needs different assumptions (multivariate normality and equality of covariance matrices) and violation of these assumptions affects the results [42].

Recently, the accessibility of powerful statistical software programs has paved the way for the application of advanced statistical models such as data mining techniques in the differential diagnosis of IDA from βTT. However, few studies have already employed such advanced statistical methods and data mining techniques for differential diagnosis of hematological data [40, 43–52]. Therefore, this study was intended to compare tree algorithms as powerful machine-learning methods and support vector machines (SVM) with hematological indices in differentiation between IDA and βTT. Tree-based methods can determine homogeneous subgroups of patients needing different treatment strategies or diagnostic tests, making these methods useful for subgroup analysis [53–56].

The tree-based methods include nonparametric methods and need no assumptions about the functional form of the data. Besides, they deal with the high-dimensional dataset, high-order interactions, and nonlinear relationships. These methods are invariant to monotone transformations of predictor variables, and are robust to outliers, missing values, and also multicollinearity. These algorithms can identify the cutoff points of important predictors to discriminate the patients. In addition, tree algorithms are easy to interpret as they display results graphically, making the results understandable without requiring statistical experience. These methods can also assist the clinician in decision making [57–62].

CART (Classification and Regression Tree) algorithm is the best-known classic tree algorithm [63], though it suffers from some problems like greediness and bias in split rule selection. Tree generating in CART is based on the greedy search algorithm, and this search cannot find a global optimum [64]. The splitting method in CART is biased toward independent variables with more distinct values [65, 66]. Several tree algorithms are proposed to solve the problems of the CART algorithm. In turn, Evtree algorithm (Evolutionary learning of globally optimal classification and regression trees) [64] has been proposed to solve the greediness problem. Tree algorithms like Quick, Unbiased and Efficient Statistical Tree (QUEST) [67], Classification Rule with Unbiased Interaction Selection and Estimation (CRUISE) [68], Generalized, Unbiased, Interaction Detection and Estimation (GUIDE) [69], Conditional Inference Trees (Ctree) [70], and Logistic Tree with Unbiased Selection (LOTUS) [62] are, in turn, suggested to solve the bias in split rule selection problem.

This study aimed to compare the diagnostic performance of the CART algorithm and remedial tree algorithms for solving the disadvantages of this algorithm and SVM with hematological discrimination indices to discriminate between IDA and βTT by using accuracy measures such as true positive rate (TPR or sensitivity), true negative rate (TNR or specificity), false positive rate (FPR), false negative rate (FNR), accuracy, Youden’s index, positive predictive value (PPV), negative predictive value (NPV), positive likelihood ratio (PLR), negative likelihood ratio (PLR), diagnostic odds ratio (DOR), F-measure, and area under the curve (AUC).

Besides, the multidimensional scaling and cluster analysis were applied to extract homogeneous subgroups of hematological discriminating indices and classification tree algorithms with a similar performance according to the accuracy measures used.

## Methods

### Sample and disease type

This study included 1178 patients with hypochromic microcytic anemia from Boghrat clinical center in Tehran, Iran. CBC analysis of EDTA-K2 anti-coagulated blood samples was performed using Sysmex kx-21 automated hematology analyzer to measure hematological parameters such as Hb (Hemoglobin), HCT (hematocrit), MCV (Mean Corpuscular Volume), MCH (Mean Corpuscular Hemoglobin), MCHC (Mean Corpuscular Hemoglobin Concentration), RBC (Red Blood Cell Count) and RDW (Red Blood Cell Distribution Width). In addition, HbA2, TIBC, serum iron and serum ferritin were measured for all patients.

### Inclusion criteria

Patients with hypochromic microcytic anemia (MCV < 80 fL, MCH < 27 pg), Hb < 12 g.dl for women and Hb < 13 g.dl for men were included in the study. Among them, 708 patients were diagnosed as βTT with HbA2 > 3.5%, and 470 patients were diagnosed as IDA with serum ferritin < 15 ng/ml according to the World Health Organization [WHO] [71, 72].

### Exclusion criteria

Patients with simultaneous presentation of both diseases, severe anemia (Hb < 8 g.dl), anemia due to chronic disease, infectious disease, chronic inflammation, pregnancy or other hemoglobinopathies were excluded.

### Statistical analysis

#### Descriptive statistics and univariate analysis

Descriptive statistics (mean, standard deviation), median and interquartile range) were evaluated for different blood parameters. Normality of data was assessed using Shapiro–wilk test. Mann–Whitney U test was also used to compare the differences between the hematological parameters of both groups (IDA and βTT). *P* < 0.05 was considered to be statistically significant.

#### Hematological discriminating indices for discriminating between IDA and βTT

Hematological indices for discrimination between IDA and βTT were computed for each patient according to their formula and cut off. These indices with their formula are shown in Additional file 1: Table S1.

#### Classification algorithms

Classification tree algorithms (CART [63], QUEST [67], CRUISE [68], J48 [73], GUIDE [69], Ctree [70], Evtree [64], C5.0 [74], and LOTUS [62]) and SVM [75] were used to discriminate IDA from βTT.

#### Accuracy measures

Diagnostic performance of discrimination indices was compared with classifications tree algorithms using accuracy measures such as sensitivity, specificity, FPR, FNR, PPV, NPV, Youden's index (sensitivity + specificity – 1), accuracy, PLR, NLR, DOR, F-measure and AUC. The discrimination method with sensitivity, specificity, PPV, NPV, Youden's index, accuracy, F-measure and AUC near to 1 provided better performance. Likewise, the discrimination method with PLR > 10, NLR < 0.1 and high DOR caused a good performance for discriminating between IDA from βTT [76, 77]. Receiver operating characteristic (ROC) curve analysis was used to compute the AUC, and compare the value of AUC of discrimination methods [78].

#### Multidimensional scaling

Multidimensional scaling method was used to create a map based on the Euclidean distance for showing similarity or dissimilarity between observations. This map can be in one dimension, two dimensions, and three dimensions or in higher dimensions. Smaller distance among two observations indicates more similar and vice versa. This used a map in two dimensions for showing similarity/dissimilarity among pairs of discrimination methods through accuracy measures such as sensitivity, specificity, PPV, NPV, Youden's Index, accuracy, PLR, NLR, F-measure, and AUC [79].

#### Cluster analysis

Cluster analysis is a method for extracting homogeneous subgroups of observations. Different algorithms are proposed for cluster analysis. This study used a complete-linkage hierarchical algorithm to determine homogeneous subgroups of methods with a similar diagnostic performance using accuracy measures. The optimal number of methods with a similar diagnostic performance was selected using 30 appropriate measures. Finally, the optimal number was selected based on the majority role [80].

### Software programs and checklists

Data analysis was done using software R 4.0.0. Package epiR and package pROC were used to compute the accuracy measures and ROC curve analysis, respectively. Classification tree algorithms like CART, J48, Ctree, Evtree, and C5.0 were fitted using packages rpart, Rweka, party, evtree, and C50, respectively. Software for tree algorithms like QUEST, CRUISE, GUIDE, and LOTUS was obtained from http://pages.stat.wisc.edu/~loh/research.html. SVM algorithm and multidimensional scaling method were fitted using package MASS and package e1071, respectively. The cluster optimal number, or homogeneous groups of diagnostic discrimination methods with a similar diagnostic performances was determined using the package of NbClust. This study was also conducted based on the Strengthening the Reporting of Observational Studies in Epidemiology (STROBE) Statement: guidelines for reporting observational studies and the Standards for Reporting Studies of Diagnostic Accuracy (STARD). These checklists can be obtained from www.equator-network.org.

## Results

This study included 1178 patients with hypochromic microcytic anemia (708 patients with βTT and 470 patients with IDA) to compare the diagnostic performance of hematological discrimination indices with classification tree algorithms and SVM, so as to discriminate IDA from βTT. Data balance was, in turn, assessed using Shannon entropy [81, 82]. Additional file 1: Table S2 indicated the descriptive statistics of hematological parameters across the type of hypochromic microcytic anemia (IDA and βTT). According to this table, all variables indicated significant difference among the groups (*P* < 0.001). CRUISE, C5.0, CART, and GUIDE algorithms can calculate the normalized importance (%) for each predictor variable. These algorithms indicated similar ranking of hematological parameters importance. In this study, the normalized importance of variables was reported based on the classification tree algorithms with the best diagnostic performance (CRUISE and C5.0 algorithms). This algorithm showed that MCV and HCT variables had the highest and lowest importance for discrimination between IDA and βTT, respectively (Additional file 1: Table S2).

**Fig. 1:**
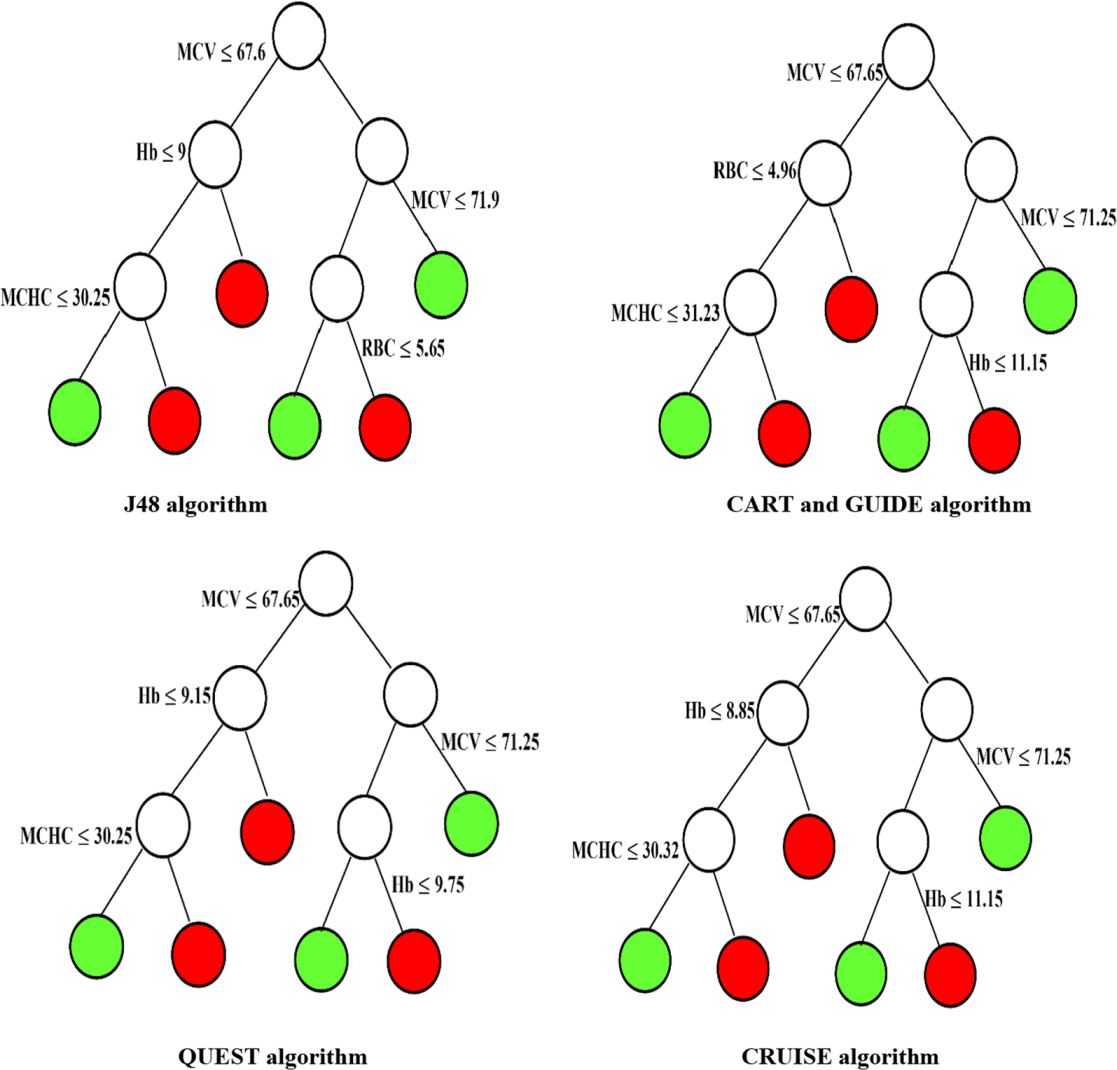
Tree structure of classification tree algorithms such as J48, CART, GUIDE, QUEST, and CRUISE (red: βeta thalassemia trait and green: iron deficiency anemia)

**Fig. 2:**
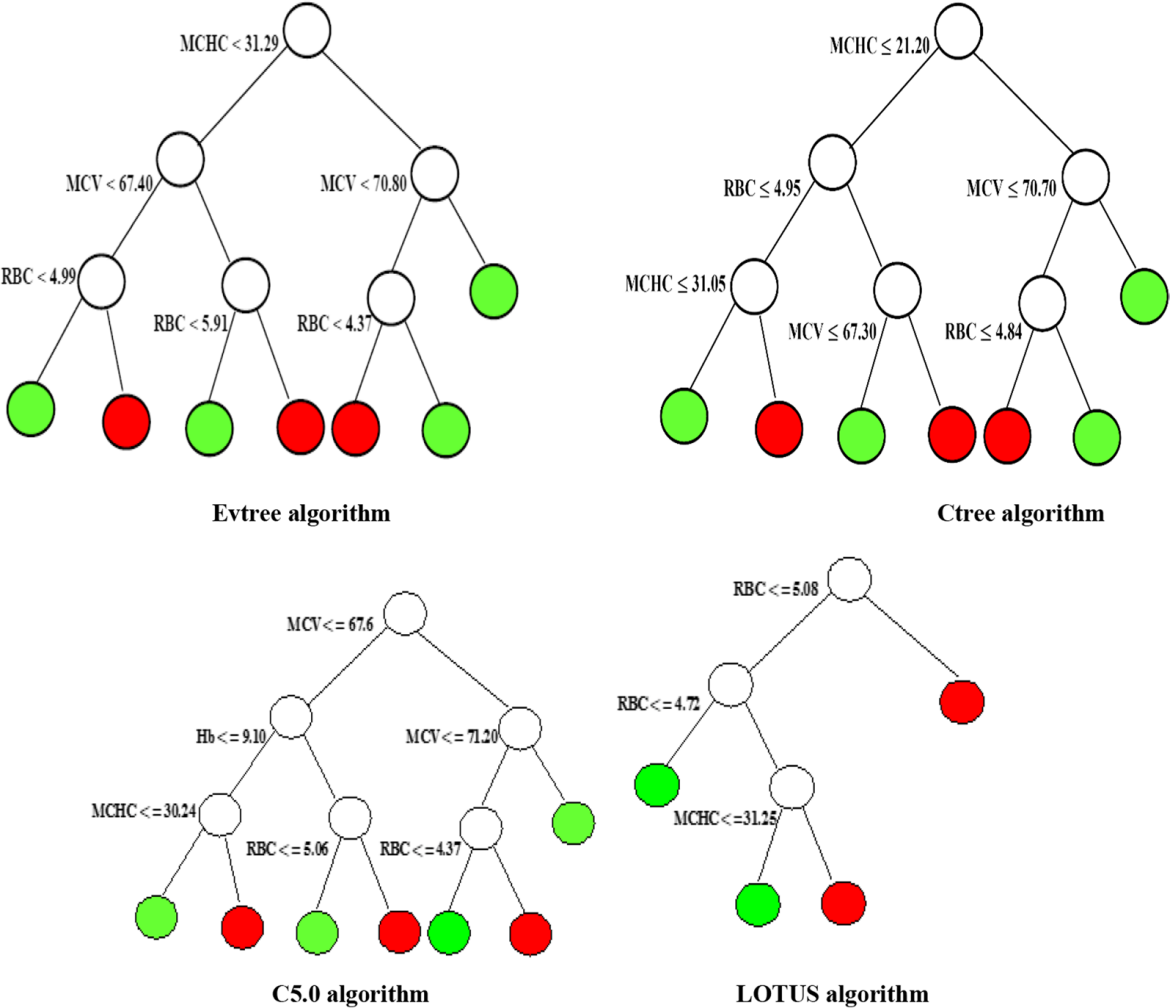
Tree structure of classification tree algorithms such as Evtree, Ctree, LOTUS, and C5.0 (red: βeta thalassemia trait and green: iron deficiency anemia)

Figures 1 and 2 indicated that all predictor variables except HCT and RDW can be used to split the nodes of tree. First variable splitting of tree-based methods except tree algorithms such as Evtree, Ctree, and LOTUS were based on the MCV with similar rule splitting. GUIDE and CART algorithms showed the same tree structure.

Additional file 1: Table S3 displays the values of accuracy measures such as sensitivity, specificity, FPR, FNR, PPV and NPV for each discrimination method (Additional file 1: Table S3).

Additional file 1: Table S3 indicated that none of the discrimination methods were fully specific for discrimination between IDA and βTT. This table showed that Janel index and CRUISE tree algorithm had the lowest FPR (while the highest TNR and PPV). In turn, the lowest TNR belonged to the Telmissani–MCHD index, while the lowest PPV was related to the Bessman (RDW) index. Shine and Lal index and Roth index showed perfect TPR (100%) and NPV (100%) as compared to other discrimination methods. Also, these indices showed the lowest FNR and the highest FPR. The lowest TPR (the highest FNR) was related to the Bessman (RDW) index, while the lowest NPV belonged to the Pornprasert (MCHC) index. All tree classification algorithms and SVM showed good performance for discriminating between IDA and βTT based on the accuracy measures like TPR, TNR, PPV and NPV in comparison to other hematological discrimination methods (Additional file 1: Table S3).

The values of accuracy measures such as Youden's index, accuracy, PLR, NLR, and DOR for each discrimination method are shown in Table 1. According to this table, the highest Youden's index/accuracy belonged to the CRUISE and C5.0 tree algorithms, while the lowest Youden's index/accuracy was for the MCHC index. Also, the highest DOR/F-measure belonged to the CRUISE and C5.0 tree algorithms, whereas the Roth index and Bessman (RDW) index had the lowest DOR/F-measure. Table 1 indicated that only CRUISE tree algorithm had PLR > 10 and discrimination methods with NLR < 0.1 were all tree algorithms except C5.0 tree algorithm and indices such as Shine and Lal, Bordbar, Sehgal, and Kerman I.

**Table 1:** Youden’s index, accuracy, positive likelihood ratio (PLR), negative likelihood ratio (NLR) and diagnostic odds ratio (DOR) of each hematological index and classification tree algorithm for differentiation between iron deficiency anemia (IDA) and β‐thalassemia trait (βTT) with their 95% confidence interval

Discriminant method	Youden's Index (%)	Accuracy (%)	PLR	NLR	DOR
CART/GUIDE	81.58 (76.26–86.05)	91.51 (89.77–93.04)	7.39 (5.83–9.37)	0.07 (0.05–0.09)	114.12 (75.09–173.43)
J48	85.12 (80.27–89.07)	93.38 (91.81–94.73)	8.41 (6.54–10.81)	0.04 (0.03–0.06)	219.56 (133.69–360.55)
QUEST	79.96 (74.43–84.65)	90.49 (88.67–92.11)	7.37 (5.79–9.36)	0.09 (0.07–0.11)	86.09 (58.24–127.27)
CRUISE	88.03 (83.52–91.59)	94.57 (93.12–95.79)	11.09 (8.28–14.86)	0.04 (0.02–0.05)	311.63 (184.41–526.62)
Ctree	81.23 (75.85–85.75)	91.26 (89.49–92.81)	7.47 (5.88–9.49)	0.07 (0.05–0.09)	105.13 (69.81–158.29)
Evtree	83.49 (78.44–87.66)	92.61 (90.97–94.04)	7.65 (6.02–9.72)	0.05 (0.03–0.07)	169.18 (106.14–269.64)
C50	87.81 (83.34–91.34)	94.65 (93.21–95.87)	9.97 (7.58–13.12)	0.03 (0.02–0.04)	374.66 (212.03–662.03)
LOTUS	39.46 (31.61–46.93)	70.97 (68.28–73.55)	2.085 (1.84–2.37)	0.38 (0.33–0.44)	5.49 (4.26–7.085)
SVM	67.93 (61.37–73.78)	84.38 (82.18–86.41)	4.76 (3.92–5.78)	0.17 (0.14–0.21)	27.86 (20.30–38.24)
England and Fraser (E&F)	48.82 (41.84–55.22)	71.65 (68.98–74.21)	5.097 (3.96–6.56)	0.44 (0.40–0.49)	11.44 (8.33–15.70)
RBC	54.89 (47.68–55.22)	79.03 (76.59–81.32)	2.80 (2.44–3.22)	0.21 (0.18–0.26)	13.28 (9.98–17.68)
Mentzer	71.25 (64.95–76.81)	86.33 (84.24–88.24)	4.99 (4.10–6.06)	0.13 (0.11–0.16)	37.66 (26.96–52.60)
Srivastava	58.83 (51.84–65.18)	78.44 (75.98–80.76)	4.74 (3.83–5.86)	0.30 (0.26–0.34)	15.70 (11.62–21.20)
Shine and Lal (S&L)	15.32 (11.66–18.90)	66.21 (63.43–68.91)	1.18 (1.14–1.23)	0	∞
Bessman (RDW)	− 15.83 (− 21.04 to − 10.61)	34.38 (31.67–37.17)	0.20 (0.13–0.30)	1.20 (1.14–1.26)	0.17 (0.11–0.26)
Ricerca	3.70 (0.04–7.52)	60.95 (58.09–63.75)	1.04 (1.01–1.07)	0.46 (0.27–0.78)	2.28 (1.31–3.97)
Green and King (G&K)	62.21 (55.29–68.47)	81.15 (78.80–83.35)	4.25 (3.52–5.13)	0.23 (0.20–0.27)	18.42 (13.68–24.81)
Das Gupta	32.87 (26.06–39.44)	71.48 (68.80–74.04)	1.56 (1.44–1.69)	0.21 (0.16–0.27)	7.52 (5.46–10.36)
Jayabose (RDWI)	57.28 (50.30–63.70)	80.64 (78.27–82.86)	2.83 (2.47–3.25)	0.17 (0.13–0.21)	17.01 (12.57–23.02)
Telmissani—MCHD	2.78 (− 0.68 to 6.40)	60.61 (57.76–63.41)	1.03 (1–1.06)	0.52 (0.30–0.90)	1.99 (1.11–3.57)
Telmissani—MDHL	40.70 (33.65–47.24)	66.81 (64.04–69.50)	4.36 (3.38–5.61)	0.54 (0.49–0.58)	8.11 (5.93–11.10)
Huber —Herklotz	6.02 (− 15.26–11.98)	46.10 (43.22–48.99)	1.47 (1.11–1.95)	0.93 (0.89–0.98)	1.58 (1.14–2.20)
Kerman I	60.66 (54.29–66.44)	83.28 (81.02–85.36)	2.77 (2.44–3.14)	0.08 (0.06–0.11)	35.83 (24.36–52.68)
Kerman II	72.96 (66.78–78.37)	86.93 (84.87–88.80)	5.63 (4.56–6.96)	0.13 (0.11–0.16)	42.01 (29.89–59.03)
Sirdah	70.86 (64.73–76.21)	84.38 (82.18–86.41)	8.57 (6.45–11.38)	0.22 (0.19–0.25)	39.28 (27.37–56.37)
Ehsani	73.38 (67.24–78.75)	87.18 (85.14–89.04)	5.67 (4.58–6.99)	0.13 (0.10–0.16)	43.85 (31.12–61.79)
Bordbar	55.05 (49.02–60.56)	81.58 (79.25–83.75)	2.29 (2.06–2.55)	0.04 (0.03–0.07)	54.87 (32.80–91.82)
Matos and Carvalho	57.27 (50.15–63.77)	77.93 (75.45–80.27)	4.20 (3.45–5.13)	0.30 (0.27–0.35)	13.89 (10.38–18.58)
Janel (11 T)	67.62 (61.41–73.08)	82.26 (79.95–84.40)	8.95 (6.63–12.07)	0.26 (0.23–0.30)	34.29 (23.75–49.50)
CRUISE Index	41.87 (34.09–49.23)	72.24 (69.59–74.78)	2.18 (1.92–2.48)	0.35 (0.30–0.41)	6.21 (4.80–8.05)
Index26	71.07 (64.87–76.50)	84.81 (82.63–86.81)	7.55 (5.81–9.81)	0.20 (0.17–0.24)	37.23 (26.29–52.72)
Hisham	51.70 (44.32–58.58)	77.25 (74.75–79.62)	2.66 (2.32–3.06)	0.25 (0.21–0.29)	10.66 (8.09–14.05)
Hameed	11.68 (5.73–17.35)	48.81 (45.92–51.71)	2.25 (1.64–3.08)	0.87 (0.83–0.91)	2.58 (1.80–3.69)
Ravanbakhsh-F1	54.11 (46.87–60.80)	78.69 (76.24–80.99)	2.74 (2.39–3.15)	0.22 (0.18–0.26)	12.74 (9.59–16.94)
Ravanbakhsh-F2	32.29 (24.46–39.83)	68.68 (65.94–71.32)	1.69 (1.53–1.88)	0.39 (0.34–0.47)	4.26 (3.30–5.50)
Ravanbakhsh-F3	50.98 (43.74–57.73)	77.76 (75.27–80.10)	2.43 (2.14–2.75)	0.21 (0.17–0.25)	11.74 (8.81–15.65)
Ravanbakhsh-F4	46.34 (39.79–52.48)	77.50 (75.01–79.86)	1.96 (1.78–2.16)	0.10 (0.08–0.14)	18.87 (12.99–27.42)
Zaghloul1	4.35 (− 3.32 to 11.86)	47.96 (45.08–50.86)	1.16 (0.97–1.39)	0.94 (0.87–1.01)	1.23 (0.95–1.59)
Zaghloul2	3.27 (− 4.43 to 10.85)	47.54 (44.65–50.44)	1.12 (0.93–1.34)	0.96 (0.89–1.03)	1.17 (0.91–1.51)
Kandhrol1	− 4.91 (− 1.31 to 3.40)	48.89 (46.01–51.79)	0.92 (0.83–1.01)	1.12 (0.98–1.28)	0.82 (0.65–1.04)
Kandhrol2	30.29 (22.67–37.66)	68.59 (65.85–71.24)	1.58 (1.44–1.74)	0.37 (0.31–0.45)	4.28 (3.29–5.57)
Alparslan	38.71 (31.29–45.79)	72.67 (70.02–75.19)	1.82 (1.65–2.02)	0.27 (0.22–0.33)	6.77 (5.13–8.94)
Merdin1	58.60 (51.48–65.09)	79.20 (76.77–81.49)	3.89 (3.25–4.69)	0.27 (0.23–0.31)	14.68 (11.01–19.59)
Merdin2	46.40 (39.10–53.16)	70.97 (68.28–73.55)	3.95 (3.18–4.90)	44.93 (40.56–49.76)	8.79 (6.57–11.75)
Roth	14.89 (11.28–18.44)	66.04 (63.26–68.75)	1.18 (1.13–1.22)	0	∞
Sargolzaie	29.79 (22.21–36.99)	61.63 (58.78–64.42)	2.57 (2.10–3.15)	0.63 (0.58–0.69)	4.07 (3.09–5.35)
Keikhaei	59.29 (52.21–65.76)	80.31 (77.92–82.54)	3.51 (2.97–4.14)	0.22 (0.19–0.26)	15.69 (11.75–20.95)
Nishad	63.96 (57.17–70.09)	82.94 (80.66–85.04)	3.81 (3.22–4.51)	0.17 (0.14–0.21)	22.16 (16.32–30.09)
Wongprachum	55.33 (48.04–62.05)	78.35 (75.89–80.67)	3.15 (2.69–3.69)	0.26 (0.22–0.30)	12.36 (9.34–16.34)
Sehgal	64.70 (58.66–70.10)	85.23 (83.07–87.21)	3.027 (2.65–3.46)	0.05 (0.03–0.07)	60.80 (38.73–95.44)
Pornprasert (MCHC)	− 32.50 (− 40 to − 24.65)	31.32 (28.68–34.06)	0.40 (0.34–0.47)	1.71 (1.54–1.90)	0.23 (0.18–0.30)
Sirachainan	9.45 (2.23–16.46)	49.58 (46.68–52.47)	1.48 (1.19–1.83)	0.88 (0.83–0.94)	1.68 (1.27–2.21)

The value of discrimination method AUC for discrimination between IDA and βTT was shown in Table 2. The ROC analysis showed that CRUISE and C5.0 tree algorithms had the highest AUC. According to the AUC, CRUISE and C5.0 tree algorithms indicated excellent diagnostic accuracy, whereas MCHC index could not be useful for discrimination between IDA and βTT. Table 2 indicated that AUC of all indices except indices such as Ricerca, Telmissani–MCHD, Huber–Herklotz, Zaghloul1, Zaghloul2 and Kandhrol1 were significantly more than 0.5, and AUC of discrimination indices such as RDW and MCHC were significantly less than 0.5 (*P* < 0.001).

**Table 2:** F-measure and AUC of each hematological index and classification tree algorithm for differentiation between iron deficiency anemia (IDA) and β‐thalassemia trait (βTT) with their 95% confidence interval

Discriminant method	F-measure (%)	AUC	Standard Error	95% CI	*P*–value
CART/GUIDE	93.04	0.908	0.009	0.891–0.925	< 0.001
J48	94.61	0.926	0.008	0.909–0.942	< 0.001
QUEST	92.12	0.889	0.009	0.882–0.918	< 0.001
CRUISE	95.54	0.940	0.007	0.926–0.955	< 0.001
Ctree	92.80	0.906	0.009	0.889–0.924	< 0.001
Evtree	93.99	0.918	0.009	0.901–0.934	< 0.001
C50	95.64	0.939	0.007	0.924–0.954	< 0.001
LOTUS	75.85	0.697	0.016	0.666–0.729	< 0.001
SVM	86.88	0.840	0.013	0.815–0.865	< 0.001
England and Fraser (E&F)	72.03	0.744	0.012	0.721–0.767	< 0.001
RBC	83.02	0.774	0.013	0.749–0.799	< 0.001
Mentzer	88.69	0.856	0.011	0.836–0.877	< 0.001
Srivastava	80.61	0.794	0.012	0.771–0.817	< 0.001
Shine and Lal (S&L)	78.06	0.577	0.008	0.560–0.593	< 0.001
Bessman (RDW)	6.76	0.421	0.009	0.401–0.440	< 0.001
Ricerca	74.89	0.519	0.007	0.505–0.532	0.281
Green and King (G&K)	83.84	0.811	0.012	0.788–0.834	< 0.001
Das Gupta	79.39	0.664	0.013	0.639–0.689	< 0.001
Jayabose (RDWI)	84.62	0.786	0.012	0.762–0.811	< 0.001
Telmissani—MCHD	74.76	0.514	0.006	0.502–0.526	0.419
Telmissani—MDHL	65.67	0.704	0.012	0.679–0.727	< 0.001
Huber—Herklotz	29.52	0.530	0.011	0.509–0.551	0.080
Kerman I	87.22	0.803	0.012	0.780–0.826	< 0.001
Kerman II	89.08	0.865	0.010	0.845–0.885	< 0.001
Sirdah	86.06	0.854	0.010	0.835–0.874	< 0.001
Ehsani	89.31	0.867	0.010	0.847–0.887	< 0.001
Keikhaei	83.49	0.797	0.012	0.773–0.820	< 0.001
Nishad	85.93	0.819	0.012	0.797–0.843	< 0.001
Wongprachum	81.83	0.777	0.013	0.752–0.801	< 0.001
Sehgal	88.72	0.824	0.011	0.801–0.846	< 0.001
Pornprasert (MCHC)	27.57	0.337	0.014	0.305–0.370	< 0.001
Sirachainan	41.07	0.547	0.013	0.523–0.572	0.006
Bordbar	86.43	0.775	0.012	0.752–0.798	< 0.001
Matos and Carvalho	80.36	0.786	0.012	0.763–0.809	< 0.001
Janel (11 T)	83.76	0.838	0.010	0.818–0.858	< 0.001
CRUISE	77.02	0.709	0.014	0.683–0.736	< 0.001
Index26	86.63	0.855	0.010	0.835–0.875	< 0.001
Hisham	81.39	0.759	0.013	0.733–0.784	< 0.001
Hameed	33.07	0.558	0.010	0.538–0.578	0.001
Ravanbakhsh-F1	82.77	0.771	0.013	0.746–0.795	< 0.001
Ravanbakhsh-F2	75.12	0.661	0.014	0.634–0.689	< 0.001
Ravanbakhsh-F3	82.42	0.755	0.013	0.729–0.779	< 0.001
Ravanbakhsh-F4	83.49	0.732	0.012	0.708–0.756	< 0.001
Zaghloul1	42.01	0.522	0.014	0.495–0.548	0.207
Zaghloul2	41.81	0.516	0.014	0.489–0.543	0.341
Kandhrol1	56.06	0.475	0.015	0.447–0.504	0.153
Kandhrol2	75.88	0.671	0.014	0.644–0.697	< 0.001
Alparslan	79.04	0.694	0.013	0.668–0.719	< 0.001
Merdin1	81.99	0.793	0.012	0.769–0.817	< 0.001
Merdin2	72.01	0.732	0.012	0.708–0.756	< 0.001
Roth	77.97	0.574	0.008	0.558–0.591	< 0.001
Sargolzaie	60.42	0.649	0.013	0.623–0.674	< 0.001

The comparison between AUC values of classification tree algorithms and hematological discrimination index with the best diagnostic performance among hematological indices (Ehsani index) showed that there was a statistically significant difference between AUC values of tree algorithms with Ehsani index (*P* < 0.05). In this regard, classification tree algorithms had significantly higher AUC than the mentioned hematological discrimination index. Also, CRUISE and C5.0 tree algorithms had significantly higher AUC than other classification tree algorithms, but there was no significant difference between AUC values of Ctree and CART algorithms (*P* > 0.05).

Overall, the results showed that CRUISE and C5.0 tree algorithms had a better performance for discrimination between IDA and βTT in comparison to all indices and other classification tree methods. CRUISE tree algorithm extracted six homogenous subgroups of patients (Fig. 1). According to the tree structure of CRUISE tree algorithm, it can be concluded that patients with MCV > 67.65 or 67.65 < MCV $\le$ 71.25 and Hb $\le$ 11.15 or MCV $\le$ 67.65 and Hb $\le$ 8.85 and MCHC $\le$ 30.32 were classified as βTT. Also, patients with 67.65 < MCV $\le$ 71.25 and Hb > 11.15 or MCV $\le$ 67.65 and Hb > 8.85 or MCV $\le$ 67.65 and MCHC > 30.32 were classified as IDA.

**Fig. 3:**
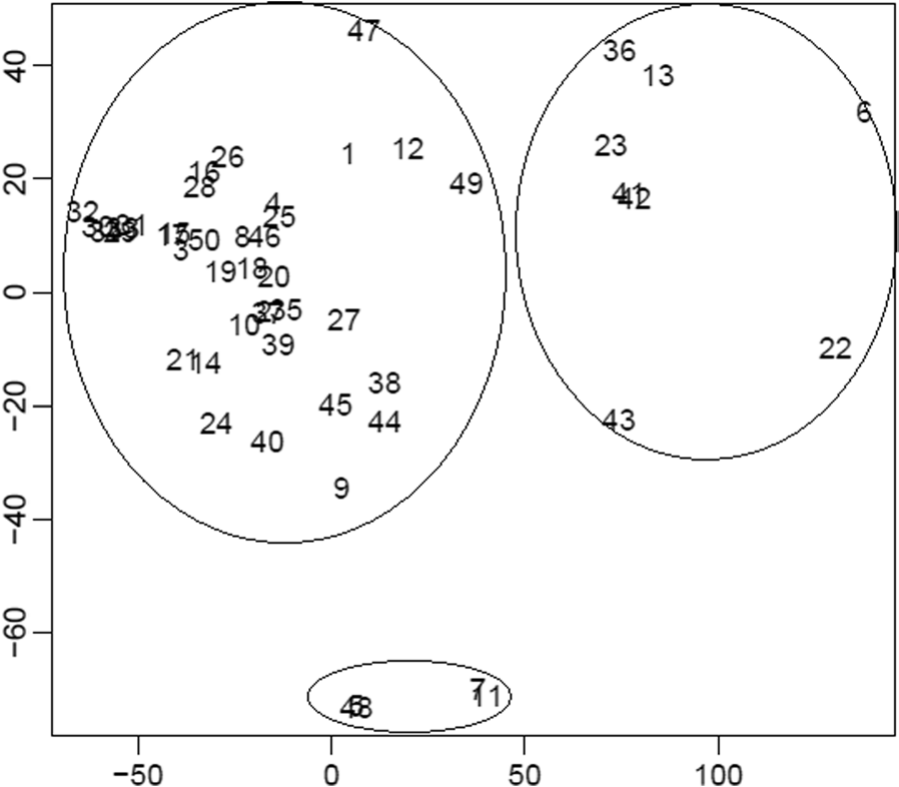
Diagram of multidimensional scaling for extracting homogeneous groups of hematological indices and classification tree algorithms with a similar diagnostic performance (1:England and Fraser, 2:RBC, 3:Mentzer, 4:Srivastava, 5:Shine and Lal, 6:Bessman (RDW), 7:Ricerca, 8:Green and King, 9:Das Gupta, 10:Jayabose (RDWI), 11:Telmissani–MCHD, 12:Telmissani–MDHL, 13:Huber–Herklotz, 14:Kerman I, 15:Kerman II, 16:Sirdah, 17:Ehsani, 18:Keikhaei, 19:Nishad, 20:Wongprachum, 21:Sehgal, 22:Pornprasert, 23:Sirachainan, 24:Bordbar, 25:Matos and Carvalho, 26:Janel (11 T), 27:CRUISE Index, 28:Index26, 29:CART/Guide, 30:J48, 31:QUEST, 32:CRUISE, 33:Ctree, 34:Evtree, 35:Hisham, 36:Hameed, 37:Ravanbakhsh-F1, 38:Ravanbakhsh-F2, 39:Ravanbakhsh-F3, 40:Ravanbakhsh-F4, 41:Zaghloul1, 42:Zaghloul2, 43:Kandhrol1, 44:Kandhrol2, 45:Alparslan, 46:Merdin1, 47:Merdin2, 48:Roth, 49: Sargolzaie, 50: C5.0, 51: LOTUS, and 52: SVM)

In addition, multidimensional scaling method extracted three subgroups of methods. The diagram of this analysis is shown in Fig. 3. One group included hematological discrimination indices such as Pornprasert, RDW, Kandhrol1, Huber–Herklotz, Sirachainan, Hameed, Zaghloul1, and Zaghloul2, while the other group included Shine and Lal, Roth, Ricerca, and Telmissani–MCHD. The third group in turn included classification tree algorithms, SVM, and some of hematological discrimination indices.

**Fig. 4:**
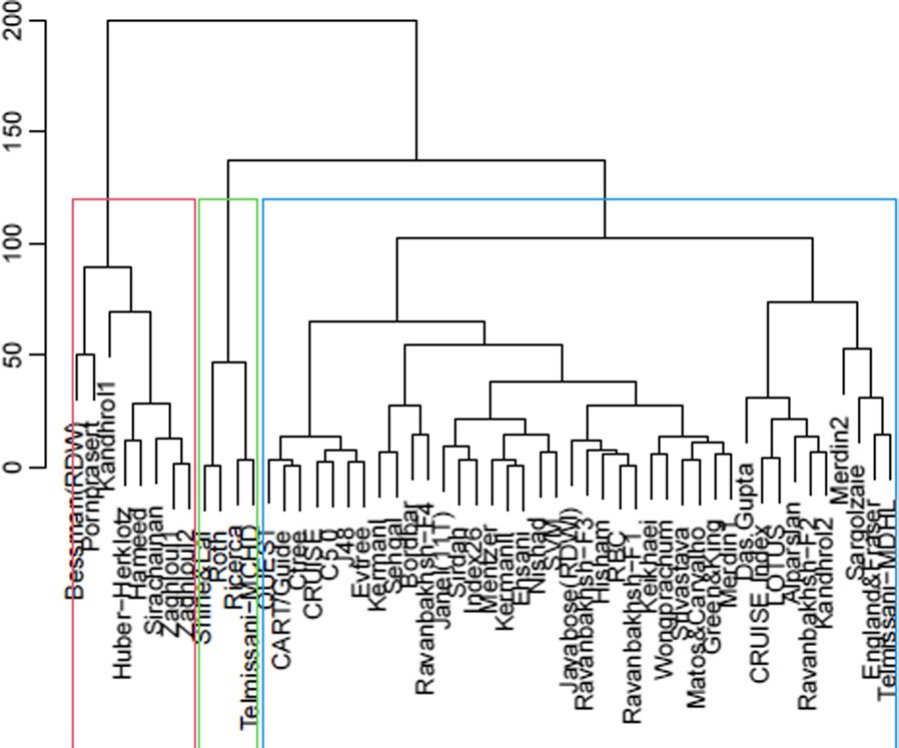
Dendrogram of cluster analysis for extracting homogeneous groups of hematological discrimination indices and classification tree algorithms with the same diagnostic performance (each rectangles includes discrimination methods with a similar diagnostic performance)

Cluster analysis like multidimensional scaling method extracted three homogenous groups of discrimination methods. The diagram of this analysis is shown in Fig. 4.

## Discussion

The two common types of microcytic anemia disorders are IDA and βTT which have similar clinical and experimental conditions [3, 11, 12]. The discrimination between these two disorders is clinically important needing time-consuming and expensive tests like HbA2, serum iron, serum ferritin and TIBC [4, 11, 13–16]. Several hematological indices are proposed for rapid and low-cost discrimination between IDA and βTT which are not fully sensitive and specific for differential diagnose [17–41].

This study used classification tree algorithms to discriminate between IDA and βTT. These are efficient and low-cost detection methods to extract homogeneous subgroups of patients [53–56]. Thus, the diagnostic performance of hematological indices was compared with tree-based methods to differentiate IDA and βTT using various accuracy measures.

Additionally, multidimensional scaling was used to extract homogeneous subgroups of methods with a similar performance based on the mentioned criteria.

The findings showed that none of the mentioned discrimination methods are fully sensitive and specific in discrimination between IDA and βTT. Also, tree-based methods exhibited high performance for differential diagnosis in comparison with the other hematological indices. CRUISE tree algorithm indicated better performance than other discrimination methods based on the amount of accuracy measures such as Youden's index, accuracy, PLR, NLR, DOR, F-measure and AUC. These criteria included both sensitivity and specificity and indicated the diagnostic performance of discrimination method more accurately than other criteria. So, this algorithm can help physicians make better clinical decision.

Although sensitivity of hematological discrimination methods such as Ricerca, Telmissani—MCHD, Bordbar, Roth, and Shine and Lal (S&L) was higher than that of CRUISE tree algorithm, these hematological indices had a high false positive rate as compared to the CRUISE tree algorithm. Moreover, with respect to the other measurements, these indices had poor performance in discriminating between IDA and βTT.

Consistent with the findings of this study, other studies demonstrated that Ehsani index had good performance in discrimination between these two disorders in comparison with other hematological indices [83, 84]. Meta-analysis studies indicated that Bessman (RDW) index had a low AUC in comparison to other hematological indices [85, 86].

Overall, the findings showed that CRUISE tree algorithm had better performance in discrimination between IDA and βTT as compared to all hematological discrimination indices and other classification tree methods. Moreover, comparison between the AUC of CRUISE and C5.0 tree algorithms and Ehsani index (this index had the best diagnostic performance in comparison to the other hematological indices) showed that there was a statistically significant difference between AUC of these discrimination methods (*P* < 0.001); CRUISE and C5.0 tree algorithms had significantly higher AUC than this discrimination index. Indeed, all accuracy measures indicated that CRUISE and C5.0 tree algorithms had the best diagnostic performance among the discrimination methods used.

Tree-based methods were fitted using hematological parameters as predictor variables. Based on the results obtained from CRUISE and C5.0 tree methods, MCV was the main hematological predictor parameter in differentiation between different types of hypochromic microcytic anemia. In this regard, it was found that the patient with βTT had lower values of MCV. In a previous study which used different decision trees for discrimination between IDA and βTT, the first split of all algorithms was based on the MCV indicating that MCV was an important predictor variable in discrimination of IDA and βTT [47].

Several studies proposed various tree-based methods for differential diagnostic between microcytic anemia [43, 44, 47, 50–52]. For instance, Bellinger et al. used classification algorithms like J48 decision tree, support vector machines (SVM), k-nearest neighbours (K-NN), multilayer perceptron (MLP) and naϊve Bayes (NB) to discriminate between patients with IDA and βTT or both [50]. In another study, Setsirichok evaluated the classification of blood characteristics by a C4.5 decision tree, a NB classifier and a MLP for classifying eighteen classes of thalassemia abnormality [43]. Likewise, Jahangiri et al. (2017) used classification tree algorithms for constructing differential scheme and investigating the performance of several tree algorithms for the differential diagnosis of IDA from βTT. In agreement with this study, Jahangiri et al. (2017) reported that CRUISE tree algorithm had the highest AUC, and MCV was an important predictor variable in the discrimination of observations into IDA and βTT, and the first split of all algorithms was based on of MCV [47]. Moreover, Chakraborty et al. (2017) utilized Ada-boost algorithm to generate multiple decision trees by using C4.5 decision tree for classification of erythrocytes or anemia detection. Their proposed approach showed accuracy, specificity and sensitivity of 97.81%, 99.7% and 97.33% respectively in detecting abnormal erythrocytes [51]. Comparing the diagnostic performance of several algorithms such as J48, K-NN, artificial neural networks and NB for identifying β-thalassemia carriers, AlAgha concluded that naϊve Bayes had the superior performance to differentiate between normal and β-thalassemia carriers [52].

Overall, the CRUISE and C5.0 tree algorithms with the best performance in this study showed better performance in comparison with tree algorithms in the previous studies [43, 87].

Using advanced methods such as tree-based methods for discriminating between IDA and βTT in addition to the differential indices can be a good idea for discriminating between these two hematologic disorders. Though each index only includes one or specific blood parameters, machine learning methods can consider the effects of all blood parameters simultaneously for data prediction and exploratory modeling. Besides, using decision trees for discrimination between IDA and βTT can avoid expensive, time‐consuming, and complicated laboratory procedures leading to non-satisfactory hematological indices in discriminating between these two hematologic disorders.

The application of methods like multidimensional scaling and cluster analysis are deemed to be useful to determine different classification methods with similar diagnostic functions. In previous hematological studies, such indices were compared subjectively based on the accuracy measures. Therefore, the application of multidimensional scaling method and cluster analysis are proposed to determine the hematological discrimination indices with similar performance for future hematological studies.

## Application in practice for medical studies

In medical diagnostic processes, decision making with high diagnostic performance is very important. Tree-based methods can be considered as appropriate methods for decision making, because they generate differential diagnosis with high accuracy measures (sensitivity, specificity, PPV, NPV, PLR, NLR, DOR, accuracy, and AUC) in comparison to the discrimination indices. In addition, tree algorithms display results graphically, making the results understandable with no statistical expertise. These algorithms can be thus useful for diagnostic classification scheme of patients in medical studies. This study thus considered the discrimination between IDA and βTT to prevent iron overload and its complications caused by misdiagnosis and inaccurate treatment, and also to determine the prenatal causes for hemoglobin chain disorders.

## Conclusions

Given its diagnostic performance, CRUISE and C5.0 tree algorithms are considered as an appropriate method for differential diagnosis of patients in comparison to other methods. Moreover, tree-based methods are useful along with other parameters for discriminating between IDA and βTT. In conclusion, considering the advantages of tree algorithms, they can help physicians make better clinical decisions. The results showed that multidimensional scaling method and cluster analysis are appropriate techniques to determine the discrimination indices with similar performance for future studies. In addition, the tree-based methods were identified as good methods for extracting homogeneous subgroups of observations in medical studies.

## Supplementary Information

**Additional file 1**. **Table S1.** Discrimination indices for differentiation between iron deficiency anemia (IDA) and β-thalassemia trait (βTT). **Table S2.** Descriptive statistics of blood parameters of the study groups and normalized importance (%) of hematological parameters based on the CRUISE tree algorithm (SD: standard deviation and IQR: interquartile range). **Table S3.** Sensitivity (TPR), specificity (TNR), false positive rate (FPR), false negative rate (FNR), positive predictive values (PPV) and negative predictive values (NPV) of each hematological index and classification tree algorithm for differentiation between iron deficiency anemia (IDA) and β-thalassemia trait (βTT) with their 95% confidence interval.

## Data Availability

The datasets used and/or analyzed during the study are available from the corresponding author on reasonable request.
