# Supplementary material for: Diagnostic performance of classification trees and hematological functions in hematologic disorders: an application of multidimensional scaling and cluster analysis
Source: BMC Med Inform Decis Mak. 2021 Nov 10;21:313. doi: 10.1186/s12911-021-01678-5 (PMC8579574; doi:10.1186/s12911-021-01678-5)
Supplement: Supplementary file 1 — Additional file 1. Table S1. Discrimination indices for differentiation between iron deficiency anemia (IDA) and β-thalassemia trait (βTT). Table S2. Descriptive statistics of blood parameters of the study groups and normalized importance (%) of hematological parameters based on the CRUISE tree algorithm (SD: standard deviation and IQR: interquartile range). Table S3. Sensitivity (TPR), specificity (TNR), false positive rate (FPR), false negative rate (FNR), positive predictive values (PPV) and negative predictive values (NPV) of each hematological index and classification tree algorithm for differentiation between iron deficiency anemia (IDA) and β-thalassemia trait (βTT) with their 95% confidence interval. [file 12911_2021_1678_MOESM1_ESM.docx]

**Table1.** Discrimination indices for differentiation between iron deficiency anemia (IDA) and β‐thalassemia trait (βTT).

| **Discriminant method** | **Reference** | **Calculation** | **Cut–off βTT** | **Cut–off IDA** |
| --- | --- | --- | --- | --- |
| **England and Fraser (E&F)** | (17) | MCV $-$ RBC $-$ (5 HB) $-$ 3.4 | < 0 | > 0 |
| **RBC** | (18) | RBC | > 5 | < 5 |
| **Mentzer** | (19) | MCV$/$RBC | < 13 | > 13 |
| **Srivastava** | (20) | MCH$/$RBC | < 3.8 | > 3.8 |
| **Shine and Lal (S&L)** | (21) | MCV^2^ $\times$ MCH $\times$ 0.01 | < 1530 | > 1530 |
| **Bessman (RDW)** | (22) | RDW | < 14 | > 14 |
| **Ricerca** | (23) | RDW$/$RBC | < 4.4 | > 4.4 |
| **Green and King (G&K)** | (24) | (MCV^2^ $\times$ RDW)$/$(100 HB) | < 65 | > 65 |
| **Das Gupta** | (25) | 1.89 RBC – 0.33 RDW –3.28 | > 0 | < 0 |
| **Jayabose (RDWI)** | (26) | (MCV$\times$RDW)$/$RBC | < 220 | > 220 |
| **Telmissani – MCHD** | (27) | MCH$/$MCV | < 0.34 | > 0.34 |
| **Telmissani – MDHL** | (27) | (MCH $\times$ RBC)$/$MCV | > 1.75 | < 1.75 |
| **Huber– Herklotz** | (28) | (MCH $\times$ RDW$/$10 RBC) + RDW | < 20 | > 20 |
| **Kerman I** | (29) | (MCV$\times$MCH)$/$RBC | < 300 | 300 – 400 |
| **Kerman II** | (29) | (MCV$\times$MCH $\times$ 10)$/$(RBC $\times$ MCHC) | < 85 | 85 – 105 |
| **Sirdah** | (30) | MCV − RBC − (3 Hb) | < 27 | > 27 |
| **Ehsani** | (31) | MCV − (10 RBC) | < 15 | > 15 |
| **Keikhaei** | (32) | (HB$\times$RDW $\times$ 100)$/$(RBC^2^ $\times$ MCHC) | < 21 | > 21 |
| **Nishad** | (33) | 0.615 MCV + 0.518 MCH + 0.446 RDW | < 59 | > 59 |
| **Wongprachum** | (34) | (MCV $\times$ RDW$/$RBC) – 10 HB | < 104 | > 104 |
| **Sehgal** | (35) | MCV^2^$/$RBC | < 972 | > 972 |
| **Pornprasert (MCHC)** | (36) | MCHC | < 31 | > 31 |
| **Sirachainan** | (37) | 1.5 HB – 0.05 MCV | > 14 | < 14 |
| **Bordbar** | (38) | $\left\vert80-\mathrm{MCV} \right\vert$ $\times$ $\left\vert27-\mathrm{MCH} \right\vert$ | > 44.76 | < 44.76 |
| **Matos and Carvalho** | (41) | 1.91 RBC + 0.44 MCHC | > 23.85 | < 23.85 |
| **Janel (11T)** | (39) | Combination of RBC, Mentzer, S&L, E&F, Srivastava, G&K, RDW, RDWI, Ricerca, Ehsani and Sirdah | $\geq$ 8 | < 8 |
| **CRUISE Index** | (40) | MCHC + 0.603 RBC + 0.523 RDW | $\geq$ 42.63 | < 42.63 |
| **Index26** | (40) | Combination of all indices except Janel (11T) index | $\geq$ 16 | < 16 |
| **Hisham** | (92) | MCH × RDW / RBC | < 67 | $\geq$ 67 |
| **Hameed** | (92) | MCH × HCT × RDW / (RBC × Hb)^2^ | < 220 | $\geq$ 220 |
| **Ravanbakhsh-F1** | (93) | MCV / HCT | < 2 | $\geq$ 2 |
| **Ravanbakhsh-F2** | (93) | RDW – 3 RBC | < 1.50 | $\geq$ 1.50 |
| **Ravanbakhsh-F3** | (93) | MCV × RDW – 100 RBC | < 600 | $\geq$ 600 |
| **Ravanbakhsh-F4** | (93) | MCV × Hb / RDW × RBC | < 10 | $\geq$ 10 |
| **Zaghloul1** | (94) | Hb + HCT + RBC | > 52.50 | $\leq$ 52.50 |
| **Continue on next page** | | | | |

**Table 1 (continued)**

| **Discriminant method** | **Reference** | **Calculation** | **Cut–off βTT** | **Cut–off IDA** |
| --- | --- | --- | --- | --- |
| **Zaghloul2** | (94) | Hb + HCT + RBC − RDW | > 37.10 | $\leq$ 37.10 |
| **Kandhro1** | (95) | RBC / HCT + 0.5 RDW | < 8.20 | $\geq$ 8.20 |
| **Kandhro2** | (95) | RDW × 5 / RBC | < 16.8 | $\geq$ 16.8 |
| **Alparslan** | (96) | log_10_ (MCH × MCHC × RDW / RBC) | < 3.34 | $\geq$ 3.34 |
| **Merdin1** | (96) | RDW × RBC / MCV | > 1.27 | $\leq$ 1.27 |
| **Merdin2** | (96) | RDW × RBC × Hb / MCV | > 14.70 | $\leq$ 14.70 |
| **Roth** | (51) | 1.45 ×(MCV − 82.8) / 10.28 + 0.66 × (MCH − 27.0) / 3.9 + 0.98 | < 0 | $\geq$ 0 |
| **Sargolzaie** | (97) | 125.6 + 44.3 RBC – 20.9 Hb – 2.5 MCV + 20.3 MCH –12.18 MCHC | < 0.50 | $\geq$ 0.50 |

**Table 2.** Descriptive statistics of blood parameters of the study groups and normalized importance (%) of hematological parameters based on the CRUISE tree algorithm (SD: standard deviation and IQR: interquartile range).

| **Hematological parameter** | $\boldsymbol{\beta}$**TT**  **(n = 708)** | | **IDA**  **(n = 470)** | | **P-value^*^** | **Normalized importance (%)** |
| --- | --- | --- | --- | --- | --- | --- |
|  | **Mean** $\boldsymbol{\pm}$ **SD** | **Median (IQR)** | **Mean** $\boldsymbol{\pm}$ **SD** | **Median (IQR)** |  |  |
| **MCV** | 61.37 $\pm$ 4.47 | 61.30 (5.98) | 71.65 $\pm$ 6.69 | 72.15 (9) | < 0.001 | 100 |
| **MCH** | 19.52 $\pm$ 1.63 | 19.40 (1.90) | 22.08 $\pm$ 2.94 | 22.30 (3.90) | < 0.001 | 59.50 |
| **MCHC** | 31.73 $\pm$ 1.64 | 31.84 (1.55) | 30.85 $\pm$ 3 | 30.70 (3.12) | < 0.001 | 48.70 |
| **Hb** | 10.93 $\pm$ 1.39 | 10.75 (1.60) | 10.62 $\pm$ 2.22 | 10.20 (2.10) | < 0.001 | 37.20 |
| **HCT** | 34.47 $\pm$ 4.65 | 33.80 (4.98) | 34.44 $\pm$ 6.41 | 32.80 (6.52) | < 0.001 | 13.40 |
| **RDW** | 16.43 $\pm$ 2.22 | 15.90 (2) | 15.99 $\pm$ 2.28 | 15.70 (3.30) | 0.001 | 17.90 |
| **RBC** | 5.61 $\pm$ 0.64 | 5.54 (0.77) | 4.79 $\pm$ 0.68 | 4.70 (0.79) | < 0.001 | 90.80 |

:* Mann-Whitney U test

**Table 3.** Sensitivity (TPR), specificity (TNR), false positive rate (FPR), false negative rate (FNR), positive predictive values (PPV) and negative predictive values (NPV) of each hematological index and classification tree algorithm for differentiation between iron deficiency anemia (IDA) and β‐thalassemia trait (βTT) with their 95% confidence interval.

| **Discriminant method** | **TPR (%)** | **TNR (%)** | **FNR (%)** | **FPR (%)** | **PPV (%)** | **NPV (%)** |
| --- | --- | --- | --- | --- | --- | --- |
| **CART/GUIDE** | 94.35  (92.39$-$95.93) | 87.23  (83.88$-$90.12) | 5.65  (4.07$-$7.61) | 12.77  (9.88$-$16.12) | 91.76  (89.52$-$93.65) | 91.11  (88.09$-$93.57) |
| **J48** | 96.61  (94.99$-$97.82) | 88.51  (85.28$-$91.25) | 3.39  (2.18$-$5.01) | 11.49  (8.75$-$14.72) | 92.68 (90.56$-$94.46) | 94.55 (91.99$-$96.47) |
| **QUEST** | 92.51  (90.32$-$94.34) | 87.45  (84.11$-$90.31) | 7.49  (5.66$-$9.68) | 12.55  (9.69$-$15.89) | 91.74  (89.47$-$93.65) | 88.58  (85.33$-$91.33) |
| **CRUISE** | 96.75 (95.17$-$97.93) | 91.28 (88.35$-$93.67) | 3.25  (2.07$-$4.83) | 8.72  (6.33$-$11.65) | 94.35 (92.42$-$95.92) | 94.91 (92.46$-$96.75) |
| **Ctree** | 93.79  (91.75$-$95.45) | 87.45  (84.11$-$90.31) | 6.21  (4.55$-$8.25) | 12.55  (9.69$-$15.89) | 91.84  (89.59$-$93.73) | 90.33  (87.24$-$92.89) |
| **Evtree** | 96.05  (94.33$-$97.36) | 87.45  (84.11$-$90.31) | 3.95  (2.64$-$5.67) | 12.13  (9.32$-$15.43) | 92.02  (89.82$-$93.87) | 93.62  (90.91$-$95.72) |
| **C50** | 97.60 (96.18$-$98.60) | 90.21  (87.16$-$92.74) | 2.40  (1.40$-$3.82) | 9.79  (7.26$-$12.84) | 93.76  (91.76$-$95.39) | 96.14  (93.90$-$97.74) |
| **LOTUS** | 75.85  (72.52$-$78.96) | 63.62  (59.09$-$67.98) | 24.15  (21.04$-$28.48) | 36.38  (32.02$-$41.99) | 75.85  (72.52$-$78.96) | 63.62  (59.09$-$67.98) |
| **SVM** | 86.02  (83.24$-$88.49) | 81.92  (78.13$-$85.29) | 13.98  (11.51$-$16.76) | 18.08  (14.71$-$21.87) | 87.75  (85.08$-$90.10) | 79.54  (75.67$-$83.05) |
| **England and Fraser (E&F)** | 60.73  (57.03$-$64.35) | 88.08  (84.81$-$90.87) | 39.26  (35.65$-$42.97) | 11.92  (9.13$-$15.19) | 88.48  (85.30$-$91.18) | 59.83  (56.07$-$63.50) |
| **RBC** | 85.31  (82.49$-$87.84) | 69.57  (65.20$-$73.71) | 14.69  (12.16$-$17.51) | 30.43  (26.29$-$34.80) | 80.86  (77.85$-$83.62) | 75.87  (71.54$-$79.84) |
| **Mentzer** | 89.12  (86.60$-$91.32) | 82.13  (78.36$-$85.49) | 10.88  (8.68$-$13.40) | 17.87  (14.51$-$21.64) | 88.25  (85.66$-$90.52) | 83.37  (79.66$-$86.65) |
| **Srivastava** | 74.58  (71.20$-$77.75) | 84.26  (80.64$-$87.43) | 25.42  (22.25$-$28.80) | 15.74  (12.66$-$19.36) | 87.71  (84.82$-$90.22) | 68.75  (64.79$-$72.52) |
| **Shine and Lal**  **(S&L)** | 100  (99.48$-$100) | 15.32  (12.18$-$18.90) | 0  (0$-$0.52) | 84.68  (81.10$-$87.82) | 64.01  (61.11$-$66.85) | 100  (95$-$100) |
| **Bessman (RDW)** | 3.95  (2.64$-$5.67) | 80.21  (76.32$-$83.72) | 96.04  (94.33$-$97.36) | 19.79  (16.28$-$23.68) | 23.14  (15.96$-$31.68) | 35.67  (32.78$-$38.64) |
| **Ricerca** | 96.89  (95.33$-$98.04) | 6.81  (4.70$-$0.09) | 3.11  (1.96$-$4.67) | 93.19  (99.91$-$95.30) | 61.03  (58.11$-$63.89) | 59.26  (45.03$-$72.43) |
| **Green and King (G&K)** | 81.36  (78.29$-$84.16) | 80.85  (76.80$-$84.31) | 18.64  (15.84$-$21.71) | 19.15  (15.69$-$23.20) | 86.49  (83.65$-$88.99) | 74.22  (70.20$-$77.96) |
| **Das Gupta** | 91.38  (89.07$-$93.35) | 41.49  (36.99$-$46.09) | 8.62  (6.65$-$10.93) | 58.51  (53.91$-$63.01) | 70.17  (67.10$-$73.11) | 76.17  (70.47$-$81.26) |
| **Jayabose (RDWI)** | 88.56  (85.98$-$90.81) | 68.72  (64.32$-$72.89) | 11.44  (9.19$-$14.02) | 31.28  (27.11$-$35.68) | 81.01  (78.06$-$83.71) | 79.95  (75.71$-$83.75) |
| **Telmissani–MCHD** | 97.03  (95.50$-$98.16) | 5.74  (3.82$-$8.25) | 2.97  (1.84$-$4.50) | 94.26  (91.75$-$96.18) | 60.80  (57.88$-$63.66) | 56.25  (41.18$-$70.52) |
| **Telmissani–MDHL** | 52.82  (49.07$-$56.55) | 87.87  (84.57$-$90.68) | 47.18  (43.45$-$50.93) | 12.13  (9.32$-$15.43) | 86.78  (83.21$-$89.83) | 55.29  (51.64$-$58.89) |
| **Huber– Herklotz** | 18.78  (15.97$-$21.86) | 87.23  (83.88$-$90.12) | 81.22  (78.14$-$84.03) | 12.77  (9.88$-$16.12) | 68.91  (61.87$-$75.36) | 41.62  (38.52$-$44.78) |
| **Kerman I** | 94.92  (93.03$-$96.41) | 65.74  (61.26$-$70.03) | 5.08  (3.59$-$6.97) | 34.26  (29.97$-$38.74) | 80.67  (77.82$-$83.30) | 89.56  (85.85$-$92.58) |
| **Kerman II** | 88.70  (86.14$-$90.94) | 84.26  (80.64$-$87.43) | 11.30  (9.06$-$13.86) | 15.74  (12.57$-$19.36) | 89.46  (86.95$-$91.63) | 83.19  (79.52$-$86.44) |
| **Sirdah** | 80.22  (77.10$-$83.10) | 90.64  (87.64$-$93.12) | 19.77  (16.90$-$22.90) | 9.36  (6.88$-$12.36) | 92.81  (90.47$-$94.73) | 75.26 (71.50$-$78.77) |
| **Ehsani** | 89.12  (86.60$-$91.32) | 84.26  (80.64$-$87.43) | 10.88  (8.68$-$13.40) | 15.74  (12.57$-$19.36) | 89.50  (87$-$91.67) | 83.72  (80.08$-$86.93) |
| **Continue on next page** | | | | | | |

**Table 3 (continued)**

| **Discriminant method** | **TPR (%)** | **TNR (%)** | **FNR (%)** | **FPR (%)** | **PPV (%)** | **NPV (%)** |
| --- | --- | --- | --- | --- | --- | --- |
| **Bordbar** | 97.60  (96.18$-$98.60) | 57.45  (52.84$-$61.96) | 2.40  (1.40$-$3.82) | 42.55  (38.04$-$47.16) | 77.55  (74.67$-$80.25) | 94.08  (90.69$-$96.51) |
| **Matos and Carvalho** | 75.14  (71.79$-$78.29) | 82.13  (78.36$-$85.49) | 24.86  (21.71$-$28.21) | 17.87  (14.51$-$21.64) | 86.36 (83.40$-$88.98) | 68.68  (64.67$-$72.50) |
| **Janel (11T)** | 76.13 (72.81$-$79.23) | 91.49 (88.59$-$93.85) | 23.87  (20.77$-$27.19) | 8.51  (6.15$-$11.41) | 93.09  (90.71$-$95.02) | 71.79  (68$-$75.36) |
| **CRUISE Index** | 77.40  (74.14$-$80.43) | 64.47  (59.95$-$68.80) | 22.60  (19.57$-$25.86) | 35.53  (31.20$-$40.05) | 76.64  (73.37$-$79.70) | 65.44  (60.92$-$69.77) |
| **Index26** | 81.92  (78.88$-$84.69) | 89.15 (85.98$-$91.81) | 18.08  (15.31$-$21.12) | 10.85  (8.19$-$14.02) | 91.92  (89.51$-$93.92) | 76.60  (72.82$-$80.09) |
| **Hisham** | 82.77  (79.78$-$85.48) | 68.94  (64.54$-$73.09) | 17.23  (14.52$-$20.22) | 31.06  (26.91$-$35.46) | 80.06  (76.97$-$82.89) | 72.65  (68.26$-$76.73) |
| **Hameed** | 21.05  (18.09$-$24.24) | 90.64  (87.64$-$93.12) | 78.95  (75.76$-$81.91) | 9.36  (6.88$-$12.36) | 77.20  (70.63$-$82.92) | 43.25  (40.13$-$46.41) |
| **Ravanbakhsh-F1** | 85.17  (82.34$-$87.71) | 68.94  (64.54$-$73.09) | 14.83  (12.29$-$17.66) | 31.06  (26.91$-$35.46) | 80.51  (77.49$-$83.29) | 75.52  (71.17$-$79.52) |
| **Ravanbakhsh-F2** | 78.67  (75.47$-$81.64) | 53.62  (48.99$-$58.19) | 21.33  (18.36$-$24.53) | 46.38  (41.81$-$51.01) | 71.87  (68.56$-$75.01) | 62.53  (57.60$-$67.27) |
| **Ravanbakhsh-F3** | 86.72  (83.99$-$89.14) | 64.26  (59.74$-$68.59) | 13.28  (10.86$-$16.01) | 35.74  (31.41$-$40.26) | 78.52  (75.47$-$81.35) | 76.26  (71.76$-$80.37) |
| **Ravanbakhsh-F4** | 94.63  (92.71$-$96.17) | 51.70  (47.08$-$56.30) | 5.37  (3.83$-$7.29) | 48.30  (43.70$-$52.92) | 74.69  (71.71$-$77.51) | 86.48  (81.91$-$90.25) |
| **Zaghloul1** | 31.36 (27.95$-$34.92) | 72.98 (68.72$-$76.94) | 68.64  (65.08$-$72.05) | 27.02  (23.06$-$31.28) | 63.61  (58.32$-$68.67) | 41.38 (37.99$-$44.81) |
| **Zaghloul2** | 31.36 (27.95$-$34.92) | 71.92  (67.62$-$75.94) | 68.64  (65.08$-$72.05) | 28.08  (24.06$-$32.38) | 62.71 (57.44$-$67.77) | 41.02 (37.64$-$44.47) |
| **Kandhrol1** | 54.24  (50.49$-$57.95) | 40.85  (36.37$-$45.45) | 45.76  (42.05$-$49.51) | 59.15  (54.55$-$63.63) | 58.01  (54.14$-$61.79) | 37.21 (33.03$-$41.54) |
| **Kandhrol2** | 82.20  (79.18$-$84.95) | 48.09  (43.49$-$52.71) | 17.80  (15.05$-$20.82) | 51.91  (47.29$-$56.51) | 70.46  (67.22$-$73.55) | 64.21  (58.95$-$69.22) |
| **Alparslan** | 85.73  (82.94$-$88.23) | 52.98  (48.35$-$57.57) | 14.27  (11.77$-$17.06) | 47.02  (42.43$-$51.65) | 73.31  (70.16$-$76.30) | 71.14  (66.09$-$75.84) |
| **Merdin1** | 78.81  (75.62$-$81.77) | 79.79  (75.87$-$83.33) | 21.19  (18.23$-$24.38) | 20.21  (16.67$-$24.13) | 85.45  (82.51$-$88.07) | 71.43  (67.36$-$75.26) |
| **Merdin2** | 62.15  (58.46$-$65.73) | 84.26  (80.64$-$87.43) | 37.85  (34.27$-$41.54) | 15.74  (12.57$-$19.36) | 85.60  (82.27$-$88.52) | 59.64  (55.79$-$63.39) |
| **Roth** | 100  (99.48$-$100) | 14.89  (11.79$-$18.44) | 0  (0$-$0.52) | 85.11  (81.56$-$88.21) | 63.89 (60.99$-$66.73) | 100  (94.87$-$100) |
| **Sargolzaie** | 48.73  (44.99$-$52.48) | 81.06  (77.22$-$84.51) | 51.27  (47.52$-$55.01) | 18.94  (15.49$-$22.78) | 79.49  (75.38$-$83.19) | 51.21  (47.55$-$54.86) |
| **Keikhaei** | 82.91 (79.93$-$85.61) | 76.38  (72.28$-$80.15) | 17.09  (14.39$-$20.07) | 23.62  (19.85$-$27.72) | 84.10  (81.17$-$86.73) | 74.79 (70.66$-$78.62) |
| **Nishad** | 86.72  (83.10$-$89.14) | 77.23  (73.17$-$80.95) | 13.28  (10.86$-$16.90) | 22.77  (19.05$-$26.83) | 85.16  (82.35$-$87.68) | 79.43  (75.43$-$83.05) |
| **Wongprachum** | 81.07  (77.99$-$83.90) | 74.26  (70.05$-$78.15) | 18.93  (16.10$-$22.01) | 25.74  (21.85$-$29.95) | 82.59  (79.56$-$85.34) | 72.26  (68.03$-$76.21) |
| **Sehgal** | 96.61  (94.99$-$97.82 | 68.09  (63.66$-$72.28) | 3.39  (2.18$-$5.01) | 31.91  (27.72$-$36.34) | 82.01  (79.24$-$84.56) | 93.02 (89.80$-$95.48) |
| **Pornprasert (MCHC)** | 21.75  (18.77$-$24.98) | 45.75  (41.17$-$50.37) | 78.25  (75.02$-$81.23) | 54.25  (49.63$-$58.83) | 37.65  (32.94$-$42.55) | 27.96 (24.81$-$31.28) |
| **Sirachainan** | 29.24  (25.91$-$32.74) | 80.21  (76.32$-$83.72) | 70.76  (67.26$-$74.09) | 19.79  (16.28$-$23.68) | 69  (63.43$-$74.19) | 42.94  (39.64$-$46.29) |
